# A rare case of eosinophilic gastrointestinal disorders with short bowel syndrome after strangulated bowel obstruction

**DOI:** 10.1186/s40792-022-01527-1

**Published:** 2022-09-14

**Authors:** Yuhki Arai, Yoshiaki Kinoshita, Takashi Kobayashi, Yoshiaki Takahashi, Toshiyuki Ohyama, Naoki Yokota, Yu Sugai, Shoichi Takano, Yu Hamasaki, Utako Kaneko, Satoshi Kanada

**Affiliations:** 1 Department of Pediatric Surgery, Niigata University Graduate School of Medical and Dental Sciences, 1-757, Asahimachi-dori, Chuo-ku, Niigata City, 951-8510 Japan; 2 Department of Pediatrics, Niigata University Graduate School of Medical and Dental Sciences, 1-757, Asahimachi-dori, Chuo-ku, Niigata City, 951-8510 Japan; 3 Department of Pediatric Surgery, Nagaoka Red Cross Hospital, 2-297-1 Sensyu, Nagaoka-city, Niigata 940-2085 Japan

**Keywords:** Eosinophilic gastrointestinal disorders, Eosinophilic colitis, Short bowel syndrome, Prednisolone

## Abstract

**Background:**

Short bowel syndrome (SBS) is a rare yet costly disease with an incidence rate of 3 per million people. Herein, we report a rare case of eosinophilic gastrointestinal disorders (EGIDs) with SBS after strangulated bowel obstruction.

**Case presentation:**

A 5-year-old male had a necrotic intestine of 340 cm resected due to strangulated bowel obstruction caused by an intestinal mesenteric hiatal hernia. The length of the residual intestine was 51 cm. Bloody stools appeared 19 days postoperatively. Colonoscopy showed diffuse redness of the colonic mucosa, and pathological findings showed moderate chronic inflammatory cellular infiltration. On blood examination, the eosinophil count was > 30%. EGIDs with short bowel syndrome (SBS) were suspected. Because his symptoms did not improve with initial nutrition therapy, he was transferred to our hospital 5 months after the operation. Prednisolone was administrated at an initial dose of 1.4 mg/kg/day, 6 days after his transfer. Bloody stools disappeared after prednisolone administration. Seven months after discharge, he had no bloody stool recurrence.

**Conclusion:**

The risk of developing secondary EGIDs in children with SBS should be considered, and postoperative management should include attention to abdominal symptoms and elevated eosinophil counts on blood examination.

## Background

Short bowel syndrome (SBS) is a rare yet costly disease with an incidence rate of 3 per million people [1]. SBS and parenteral nutrition (PN) are closely associated with chronic enteritis. A previous study has shown that inflammatory bowel disease (IBD) like symptoms and pathologic changes were found in pediatric patients with SBS, and that an etiological connection exists between SBS and chronic intestinal inflammation [2].

Here, we report a rare case of eosinophilic gastrointestinal disorder (EGIDs) with SBS after strangulated bowel obstruction.

## Case presentation

**Fig. 1:**
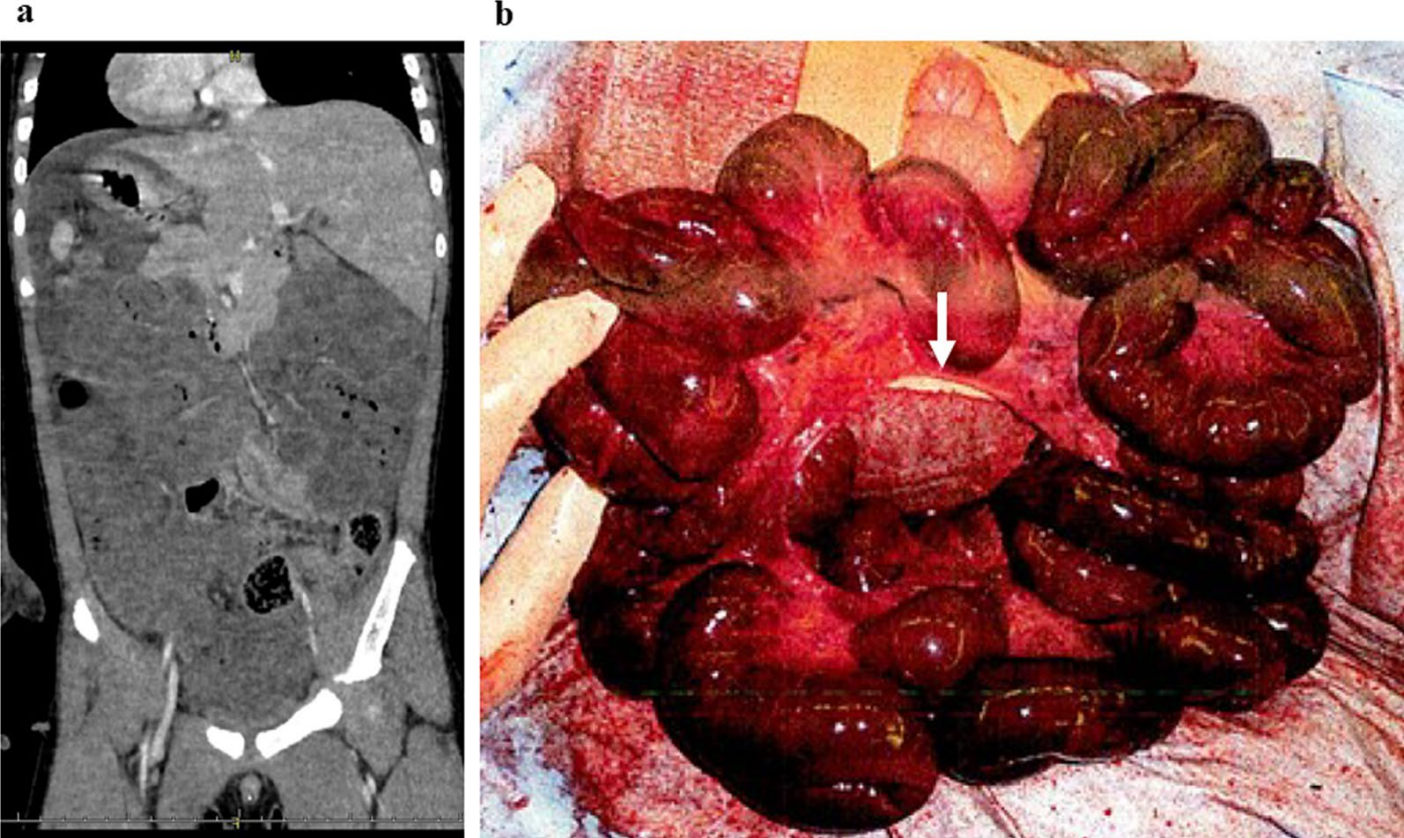
Preoperative CT and intraoperative findings. **a** Preoperative CT: a poor contrast effect on the small intestine. **b** Intraoperative findings: strangulated bowel obstruction caused by an intestinal mesenteric hiatal hernia. White arrow: intestinal mesenteric hiatal hernia

**Fig. 2:**
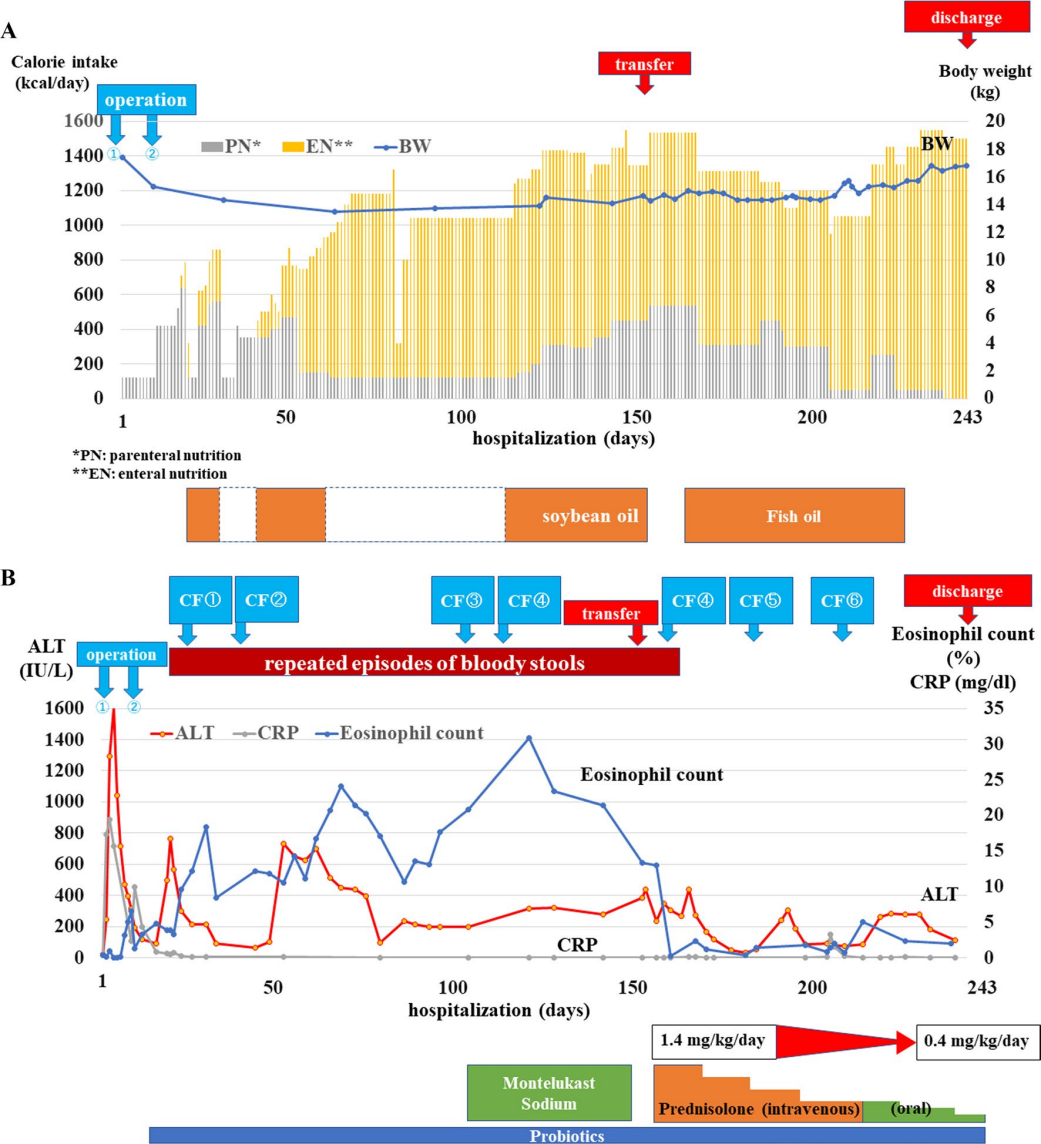
Clinical course and therapeutic management. **a** PN, EN, and body weight changes throughout the clinical course. **b** Colono fiberscope (CF), change of bloody stool, eosinophils, CRP, ALT throughout the clinical course

**Fig. 3:**
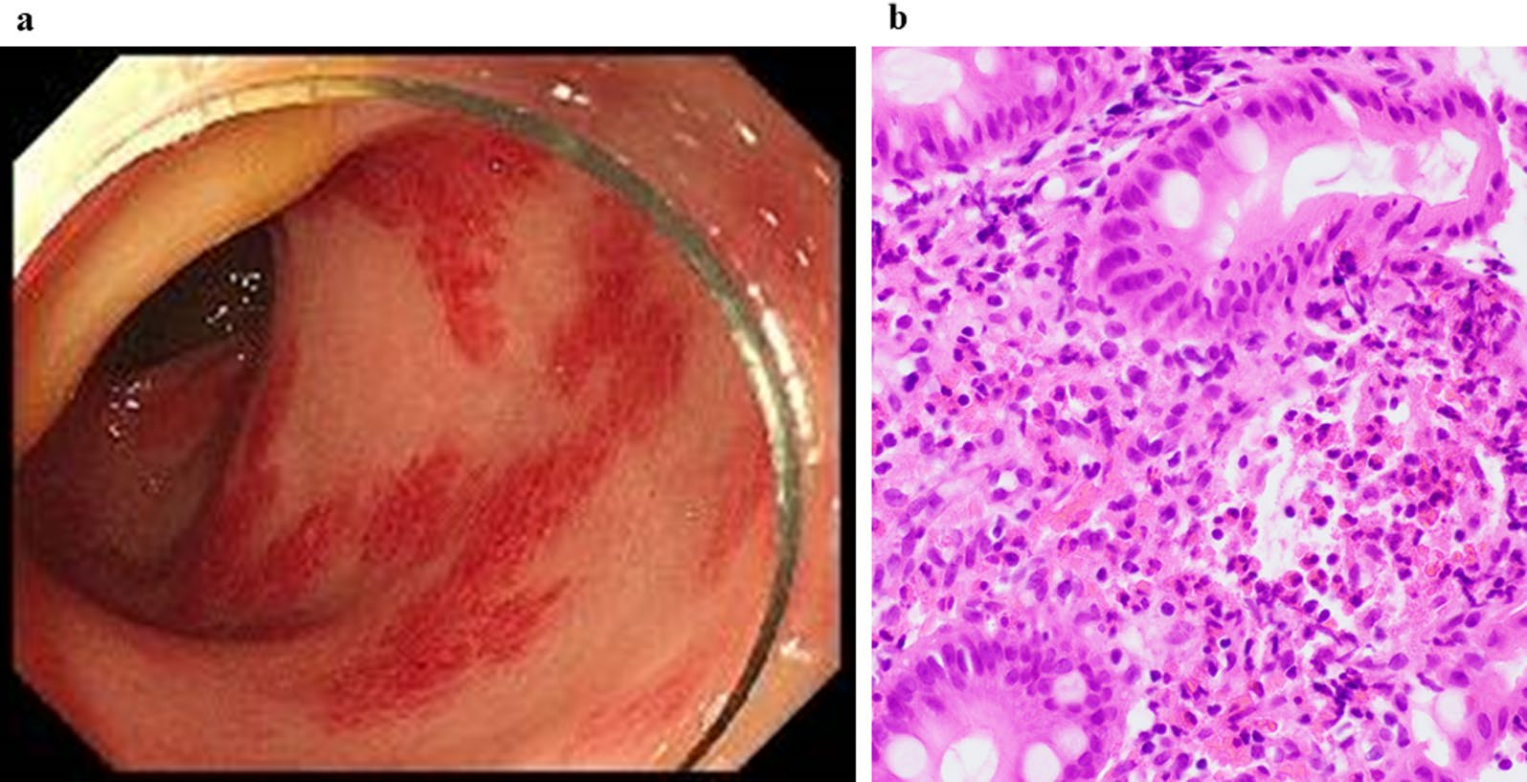
Findings prior to prednisolone administration. **a** Colonoscopy findings: diffuse redness and edema of the colonic mucosa. **b** Pathological findings of biopsy from colonoscopy: moderate inflammatory cell infiltration and numerous eosinophil counts, > 20 cells/HPF

A 5-year-old male was taken to a nearby clinic with complaints of abdominal pain and vomiting. Blood examination showed an elevated inflammatory response, progressive acidosis, and acute renal dysfunction; Computed tomography (CT) findings revealed a poor contrast effect on the small intestine, with extensive necrotic changes (Fig. 1a). He was diagnosed with strangulated bowel obstruction and underwent emergency operation. The necrotic intestine (340 cm) was resected because of strangulated bowel obstruction caused by an intestinal mesenteric hiatal hernia (Fig. 1b). The length of the residual intestine was 51 cm. His respiratory and circulatory status stabilized and inflammatory findings improved after the operation. Stoma closure was performed 8 days postoperatively. His general condition remained stable without any complications; however, bloody stools appeared 19 days after the first operation (Fig. 2a, b). No pathogenic bacteria were detected in the stool at the time of bloody stool appearance. The patient underwent esophagogastroduodenoscopy and colonoscopy. Diffuse redness and edema were observed in the colonic mucosa throughout the colon (Fig. 3a). In contrast, no inflammatory findings were observed in the upper gastrointestinal tract. Pathological findings in the colonic mucosa showed moderate inflammatory cell infiltration and numerous eosinophil counts, > 20 cells/high-power field (cells/HPF) (Fig. 3b). A blood examination revealed an eosinophil count of more than 20%. The patient was diagnosed with EGIDs. The treatment for SBS was considered effective as the EGIDs treatment. Nutritional therapies, which comprised PN using fat and amino acid formulas and enteral nutrition (EN) using elemental and low residue diet, were initiated. A second colonoscopy 9 days later revealed that the colonic mucosal erosions and edema tended to improve in response to the nutritional and medication therapy, which also included probiotics. However, he experienced repeated episodes of bloody stools that disappeared and flared up. Furthermore, liver dysfunction due to long-term parenteral nutrition was observed. Although the patient was complicated with liver dysfunction and eosinophilic inflammation, the EN was continued in order to prioritize the prevention of liver dysfunction progression, based on a comprehensive assessment of his blood parameters and general condition. A third colonoscopy showed worsening colonic mucosal erosions and edema. Additional treatment with montelukast sodium was initiated, however, the bloody stools persisted. He was transferred to our hospital to treat EGIDs 5 months after the first operation (Fig. 2a, b).

**Fig. 4:**
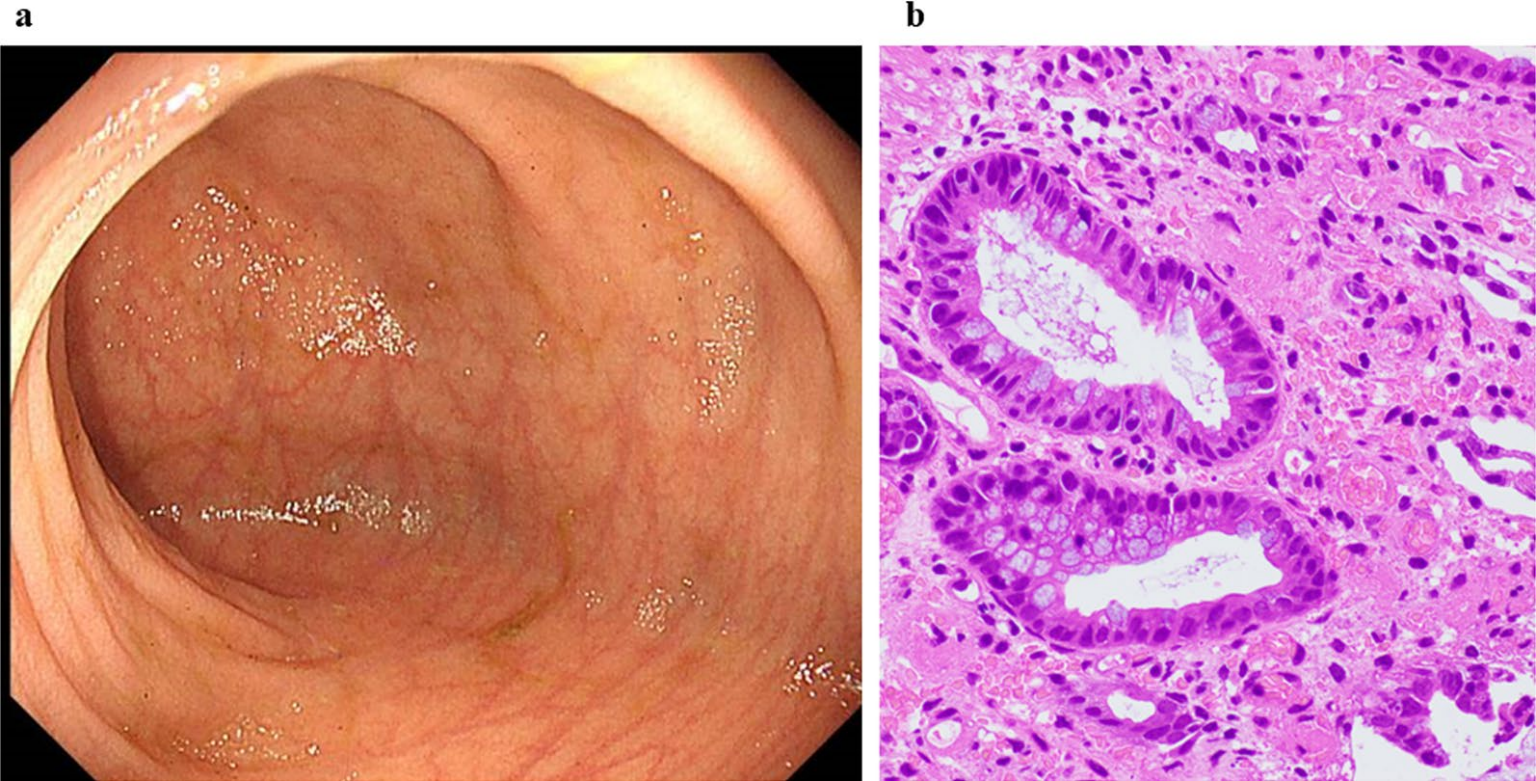
Findings after prednisolone administration. **a** Colonoscopy findings: disappearance of the redness and edema of the colonic mucosa. **b** Pathological findings of biopsy from colonoscopy: improvement of inflammatory cell infiltration and numerous eosinophil counts, 1–10 cells/HPF

At our hospital, he was managed with parental nutrition, including fish oil (Omegaven^®^; Fresenius Kabi), and EN, including an elemental diet (Fig. 2a). Prednisolone was administered at a 1.4 mg/kg/day dose 6 days after the transfer (Fig. 2b). The bloody stools disappeared a few days after prednisolone administration. The prednisolone dose was then reduced by approximately 0.2 mg every 2 weeks based on symptoms. The quantity of the elemental diet was gradually increased. As symptoms improved, dietary restrictions were lifted in a step-wise approach, including starting an allergic diet. At 85 days after the transfer, nutritional management was established without parenteral nutrition. Colonoscopy after prednisolone administration showed that the redness and edema of the colonic mucosa had disappeared (Fig. 4a). Pathological findings showed that inflammatory cell infiltration improved, and eosinophil counts were 1–10 cells/HPF (Fig. 4b). He was discharged 92 days after transfer (8 months after the operation). At the time of discharge, the prednisolone dose was reduced to 0.4 mg/kg/day. Prednisolone therapy was completed 5 months after discharge (13 months after the operation). Seven months after discontinuation of the medication, the patient had no recurrence of symptoms.

## Discussion

EGIDs are disorders of the gastrointestinal tract that result from abnormal accumulation of eosinophils in localized areas of the tract [3]. According to the inflamed organs, it is classified as eosinophilic esophagitis, eosinophilic gastritis (EG), eosinophilic gastroenteritis (EGE), and eosinophilic colitis (EC). EG and EC are often included in EGE. EGE is a rare disease that is difficult to diagnose in children.

Patients with EGE are typified by abdominal pain and diarrhea symptoms, high peripheral eosinophil counts, and gastrointestinal wall thickening, identifiable on CT [4]. Histological features of EC show numerous eosinophil counts, > 50 cells/HPF in the right colon and 30 cells/HPF in the left/transverse colon [5]. Stamm et al. reported a high prevalence of gastrointestinal eosinophilic inflammation in a large cohort of children with intestinal failure (IF) [6]. In this current case, eosinophilic inflammation was observed on blood and pathological examinations. Serum proinflammatory cytokines due to chronic inflammation associated with SBS were significantly higher in patients with SBS receiving PN, regardless of the duration of PN [7]. Although there are many theories regarding the mechanism of dietary protein allergy and systemic immune dysregulation in children with IF [8, 9], one potential triggering mechanism for allergic gastrointestinal disease in IF is increased intestinal permeability [10]. In our case, SBS after strangulated bowel obstruction operation caused the development of secondary EGIDs, similar to the mechanism. The management of patients with IF should include awareness of this typical endoscopic finding and careful screening for symptoms such as hematochezia [6].

Fundamentally, the treatment strategy for secondary EGIDs is to treat the primary disease; however, it was difficult to improve the digestive symptoms using only nutritional therapy in our case. Several studies have reported montelukast sodium as an effective treatment for EGIDs [11, 12]. Montelukast is a selective and competitive antagonist of the cysteinyl leukotriene receptor. Leukotrienes are lipid mediators released by eosinophils and are potent and selective chemoattractant for eosinophils [13, 14]. Montelukast sodium has become the focus of attention in the treatment of EGIDs. One study described the successful use of montelukast as a steroid-sparing agent in 63% adults patients [11], however its effectiveness as a treatment for EGIDs in children remains to be evaluated. In our case, sufficient effectiveness was not observed.

Glucocorticoids have been effective as an alternative treatment for EGIDs. Their efficacy stems from their ability to modulate immune responses and to inhibit the trafficking of inflammatory cells to the intestine [14]. The choice of glucocorticoids should be individualized in children with SBS-related chronic intestinal inflammation. One study reported that some pediatric patients with significant eosinophils in their intestinal biopsy responded well to glucocorticoids [15]. Thus systemic glucocorticoids can be useful for treating EGIDs.

Prednisolone activates glucocorticoid receptors and is clinically used to treat inflammatory and autoimmune disorders. We used prednisolone as one of the standard treatments for EGIDs. Prednisolone dose is often started at 0.5–2 mg/kg/day and tapered off after 1–2 weeks. Although most cases show temporary improvement, approximately 60% of patients experience recurrence of symptoms after treatment [6]. Prednisolone administration was effective in combination with other EGIDs treatment approaches. Continued follow-up with attention to symptom recurrence is necessary.

## Conclusion

The risk of developing secondary EGIDs in children with SBS should be considered. Postoperative management should focus on abdominal symptoms such as abdominal pain, bloody stools, and elevated eosinophil counts on blood examinations.

## Data Availability

Not applicable.

## Abbreviations

cells/HPF: Cells/high-power field
CF: Colono fiberscope
CT: Computed tomography
EGIDs: Eosinophilic gastrointestinal disorders
EN: Enteral nutrition
EG: Eosinophilic gastritis
EGE: Eosinophilic gastroenteritis
EC: Eosinophilic colitis
GI: Gastrointestinal
IF: Intestinal failure
IBD: Inflammatory bowel disease
SBS: Short bowel syndrome
PN: Parenteral nutrition
